# CATCH-ON Connect: A Program to Increase Technology Access Among Older Adults During COVID-19

**DOI:** 10.1093/geroni/igab046.1028

**Published:** 2021-12-17

**Authors:** Erin Emery-Tiburcio, Janis Sayer, Robyn Golden, Salvador Castaneda, Michelle Newman

**Affiliations:** 1 Rush University, Chicago, Illinois, United States; 2 Rush University Medical Center, Chicago, Illinois, United States

## Abstract

During the COVID-19 pandemic, technology became an essential tool to maintain connections to social support, health professionals, and services. However, many older adults do not have access to technology or do not feel comfortable using it. CATCH-ON Connect provides cellular-enabled tablets and individual, personalized technical assistance to older adults. Adults age 65+ in project partner primary care clinics who do not have an internet-ready device or who lack digital literacy are eligible to participate. Older adults learn how to access their electronic health record portal, use pre-installed apps (e.g., Lyft, Zoom), and receive education about COVID-19 and the 4Ms. Of the 40 participants enrolled to date, 46% have never accessed the internet with a tablet or smartphone. Initial qualitative outcomes indicate high satisfaction and increased electronic socialization. Quantitative results of participant technology challenges, loneliness, and utilization of telehealth services will be discussed.

